# How mammals stay healthy in nature: the evolution of behaviours to avoid parasites and pathogens

**DOI:** 10.1098/rstb.2017.0205

**Published:** 2018-06-04

**Authors:** Benjamin L. Hart, Lynette A. Hart

**Affiliations:** School of Veterinary Medicine, University of California, Davis, CA 95616, USA

**Keywords:** parasites, pathogens, grooming, herbal medicine, licking

## Abstract

Mammals live and thrive in environments presenting ongoing threats from parasites in the form of biting flies, ticks and intestinal worms and from pathogens as wound contaminants and agents of infectious disease. Several strategies have evolved that enable animals to deal with parasites and pathogens, including eliminating away from the sleeping–resting areas, use of an array of grooming techniques, use of saliva in licking, and consuming medicinal plant-based compounds. These strategies all are species-specific and reflect the particular environment that the animal inhabits.

This article is part of the Theo Murphy meeting issue ‘Evolution of pathogen and parasite avoidance behaviours’.

## 1. Introduction

From biting flies and ticks, to intestinal worms, to wound contaminants and to infectious diseases, animals living in nature are in an environment that presents an ongoing threat to health and even survival from the standpoint of parasites and pathogens. With the prevalence of medicine in the everyday lives of humans and their domesticated animals, it is a challenge to imagine how animals, and even prehistoric humans, living in nature with no access to vaccinations, antibiotics, worm treatments, wound medicine and the like, survive, and even thrive, as they do. The theme of this paper is to portray a sampling of behavioural adaptations that have evolved in various species and contexts to reveal how mammals do as well as they do in nature using evolved behavioural strategies to avoid and reduce infection by parasites and pathogens.

A discourse on how animals stay healthy in nature could cover a wide variety of vertebrate species, and even some non-vertebrate species, given recent research. However, staying with mammals illustrates the broad range of parasite–pathogen avoidance strategies in a group of animals with which readers are familiar. In addition, several of the avoidance strategies have been studied in greater detail in domestic mammals than is possible in the wild mammalian counterparts, lending a wider perspective to the mammals living in nature. Some aspects of this paper have been presented previously [1,2]. This review will be divided into sections that first focus on behavioural avoidance and removal of parasites and then move onto behavioural avoidance and removal of pathogens. It should be emphasized that the intention is to cover a broad sampling of the avoidance and removal strategies. There is not the space to cover all of the many examples of avoidance and removal.

One overriding concept that comes through is that animals living in nature are nowhere as free of parasites and pathogens as we might expect from looking at domestic animals. For external parasites such as fleas and ticks, and for intestinal parasites such as roundworms and tapeworms, there is a manageable parasite load in most cases that presumably does not impact fitness in a major way. Almost all defensive behaviours are carried out at some costs, such as reduced vigilance for predators or loss of feeding time; hence, having a manageable parasite load is adaptive in nature, representing a balance between parasite load and other physiological demands. This perspective is different from that in modern medicine, where administration of drugs and treatments carries no implications for reduced predator avoidance or food accessibility.

The evolution of avoidance and removal strategies for pathogens and parasites implies that the strategies evolved when there were survival and fitness pay-offs. The particular environment where an animal lives may present a particular parasite/pathogen threat that does not occur in other environments. Thus, for the widespread African antelope, the impala, discussed below, which typically inhabits the interface between wooded areas and grasslands as a defence against predators, ticks are very prevalent, and tick removal by tooth combing is very prominent. Some small rodents and felids, on the other hand, spend most of their time in dens and nearby rest areas where there is less risk from ticks but where fleas multiply by jumping back and forth between the den and rest area and the hosts’ pelage. Small felids have an evolved flea removal system, relying on systematic grooming with the tongue flea-combing morphology. For the dusky-footed wood rat, which typically finds a secure nest in a clump of sticks at the base of a bay leaf tree, fleas are also a major threat. However, the wood rats have evolved a different flea control strategy, that of bringing in fresh aromatic bay leaves that repel adult fleas and kill flea larvae that manage to hatch from ova.

## 2. Avoidance and removal strategies for biting flies

Biting flies can be more than just an annoyance. Several studies have revealed the considerable costs of flying parasites. Tabanid flies, common in Asia, East Africa and the USA, offer a good example. A study on horses found that on a typical day a horse may be bitten by about 4000 tabanid flies, accounting for a loss of as much as 0.5 l of blood [3]. In addition to causing loss of blood, flying insects also can transmit diseases.

One common means of avoiding such flies is grouping, where the *per capita* encounter with flies is diluted, the so-called encounter-dilution effect [4]. Ungulates have a variety of fly-repelling behaviours including ear twitching, head-tossing, leg stamping, muzzle flicking, muscle twitching and tail switching. As indicated in cattle, when biting fly intensity is high, fly-repelling behaviours increase and those that engage in the most fly repelling have the fewest biting flies around them [5–7].

**Figure 1.**
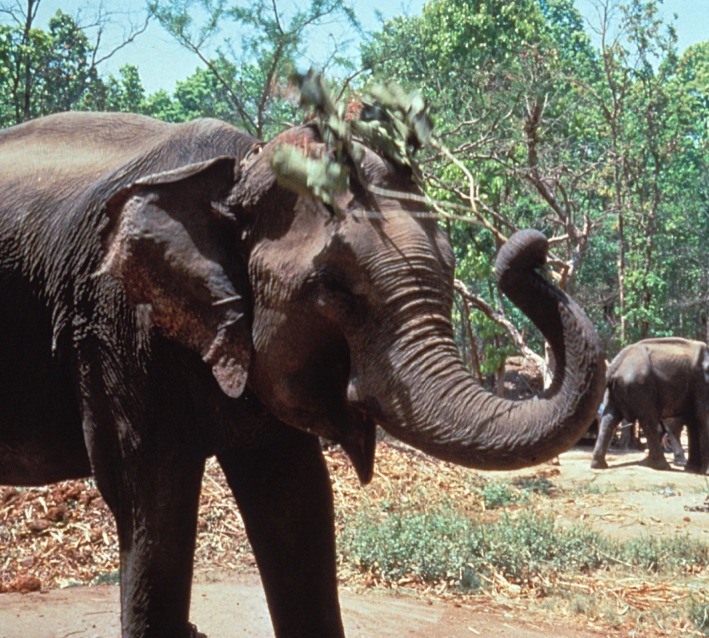
Fly switching by elephants. Biting flies are significantly reduced by this behaviour of fly switching [8]. (From [2], copyright B.L.H.). (Online version in colour.)

While on most of the body elephants have fly-resistant thick skin that large tabanids cannot penetrate, they have thinner skin on the belly, in the axillae and behind the ears, and the flies target those sites. As mentioned, flies can remove considerable blood and be costly to the animal. While the trunk would be hopeless at swatting flies, Asian elephants have evolved a way of avoiding the biting flies with a fly switch consisting of a tree branch that allows the elephant to repel flies from the belly, axillae and around the ears (figure 1). In an experiment on captive Asian elephants in Nepal, comparing the number of flies on the sides of the elephants when a branch was available to use as a fly switch with the number of flies when no branch was available, fly numbers were reduced by 43% [8]. In other experiments, when the available branch was too long or bushy, the elephants modified the branch into one of suitable size for a switch [9].

## 3. Avoidance and removal strategies for ticks

Ungulates are particularly vulnerable to ticks as they graze in grasslands. Engorging pregnant female ticks can remove considerable blood. Studies on growing cattle, for example, have revealed that a single engorging tick can reduce the annualized weight gain of a growing calf by 3 kg [10,11].

**Figure 2.**
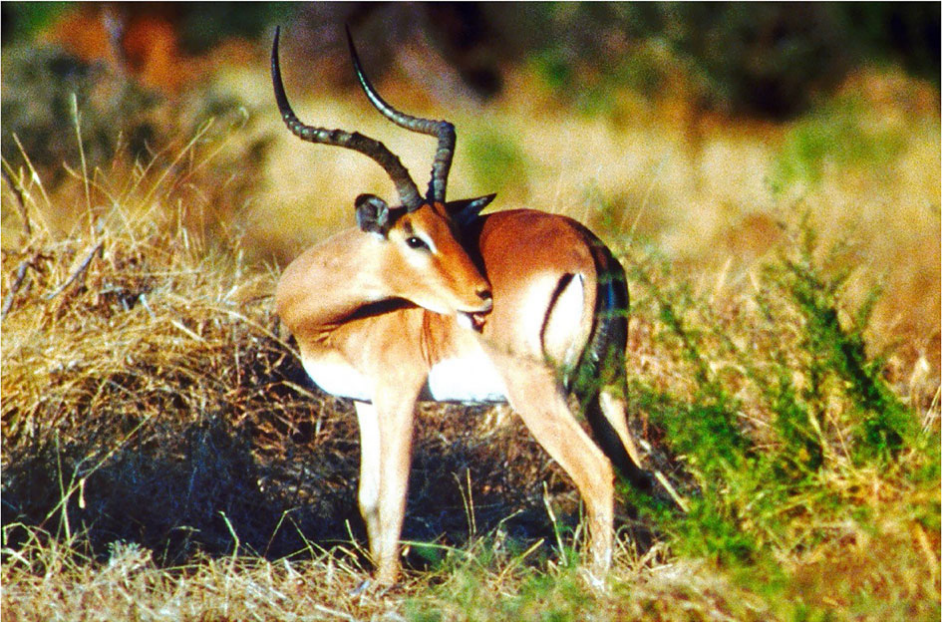
Typical grooming action of impala by swiping the tooth comb across the flank and effectively removing ticks [13]. (Copyright B.L.H.). (Online version in colour.)

For most ungulates, the first attempt at avoiding ticks is to swipe the tongue or lower incisors across the shoulders and trunk as the ticks crawl up legs and across the shoulders on the way to the head and neck or hindquarters, where they cannot be removed by oral grooming. Grooming has been especially studied in antelope of eastern and southern Africa. During grooming bouts, antelope swipe the lateral incisors across the shoulders, abdomen and flanks, presumably catching most of the traversing ticks [12]. These lower incisors make a sort of tooth comb (figure 2), which increases the effectiveness in removing adult ticks [13] (figure 2). In one experiment where impala were prevented from grooming the body by a harness for three weeks, the harnessed impala ended up with 20 times more adult and engorged ticks than the impala with control harnesses that could still groom [14]. When the harnesses were removed, and the impala went back to grooming, the frequency of post-harness grooming was 2.5 times more than that of the control impala, removing the build-up of ticks.

Much of the grooming seen in antelope and other ungulates is referred to as programmed grooming, in which bouts of oral grooming are delivered to the body regularly, driven by an envisioned underlying central mechanism [12]. This mechanism is influenced by several factors, of which body size is important. Antelope of large body size, like the eland, which lose fewer resources per body surface area to feeding ticks than smaller sized antelope, groom less frequently and correspondingly have a higher density of ticks per unit of surface area [15].

The environmental threat of tick exposure can influence the programmed grooming rate. For example, the dwarf antelope, steinbok, inhabits areas flush with vegetation and ticks. Another dwarf antelope of similar size, the klipspringer, inhabits rocky outcroppings that support few ticks. One would predict that the steinbok would have evolved a faster-paced programmed grooming rate than would have klipspringers. This was tested in a tick-free zoological environment where members of the two antelope species lived in adjacent enclosures. Klipspringers delivered oral grooming bouts at just 11% of the hourly rate of the steinbok [16,17].

The programmed grooming rate is also influenced by testosterone. In breeding male impala, vigilance over the females appears to reduce grooming time to about one-fourth that of the females. Correspondingly, males were found to carry about six times the tick load of females [18]. Two related experiments on goats reveal more about the hormonal control of the central programmed grooming mechanism. In an environment with no ectoparasites, gonadally intact adult male goats groom significantly less than females. Castration of the males is followed by an increase in grooming so that males deliver grooming bouts at the same frequency as females [19]. When castrated male goats were given testosterone, they then groomed less frequently than did females [20].

**Figure 3.**
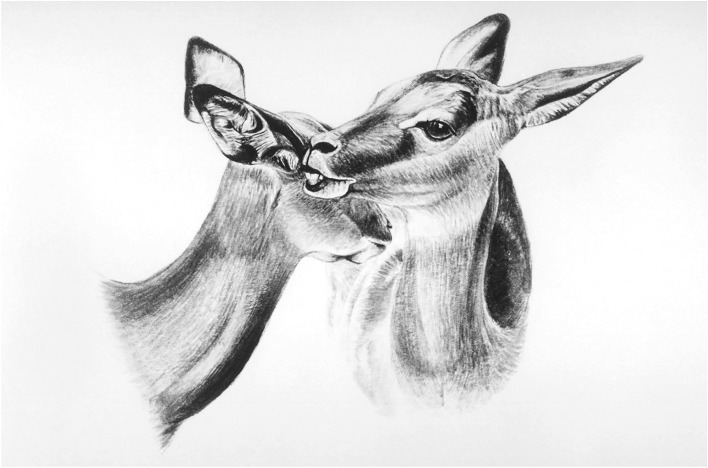
Reciprocal allogrooming by impala. In a typical encounter, the impala exchange six to eight grooming bouts [21]. (Copyright B.L.H.). Drawing by Emma Mooring.

In response to the ongoing threat from ticks reaching the head and neck area, where impala cannot remove them by self-grooming, impala have evolved a reciprocal allogrooming system of exchanging grooming bouts with another impala in a tit-for-tat manner that is unique among antelope. One impala approaches another in the group, often not related, and directs a bout of grooming episodes to the head or neck of the other. The partner typically reciprocates with an equivalent bout of grooming (figure 3). This tit-for-tat exchange of grooming bouts continues for six to 12 exchanges [21] (figure 3). If the impala approached does not reciprocate after receiving a bout or two, the initiator walks away. One trade-off for reciprocal allogrooming is the distraction from vigilance for predators during exchanging bouts. Field tests reveal that during an allogrooming encounter the partner doing the grooming is significantly slower to notice a potential predator than is the partner receiving the grooming at the time [22].

The evolved innate basis of this reciprocal allogrooming system is evident in observations of newborn impala fawns where they can be observed in a tick-free zoological setting. These fawns exchanged bouts of grooming episodes with their dam or other females, and attempted to reciprocally allogroom with other fawns in the first week after birth. Even orphaned impala fawns raised with gazelles (which do not allogroom) repeatedly tried to initiate reciprocal grooming with the gazelles even though the gazelles never returned any grooming [23].

Non-human primates, such as baboons and rhesus monkeys, have been studied extensively with regard to allogrooming, or social grooming, and the role in maintaining affiliative relationships. It has been estimated that some non-human primates invest at least one-fifth of their time in grooming [24]. However, until just recently the study of primate grooming was focused just on the social interactions and the grooming partners. This emphasis is understandable because the studies often take place in parasite-free enclosures, or involve wild primates, where examining for ectoparasites would not be feasible. In a study on yellow baboons by a research group in the Amboseli region of East Africa, data were obtained on both tick load and distribution of social grooming among group members. Investigators reported that younger and higher-ranking adults were groomed more frequently than older, low-ranking adults, and females were groomed more often than males. The baboons that received the most grooming had lowest tick loads [25]. The baboons that received the least grooming were the most likely to have wounds that could be infected and lead to fatal consequences. The animal receiving the least grooming also had the lowest measures of packed red cell volume, which is related to anaemia.

This research on baboons may provoke other studies on various species of primates where social grooming is prominent. In nature, self-grooming and social grooming are the main ways of avoiding detrimental tick infestation in primates as in other frequently grooming species. For primates, it appears as though social grooming can have health consequences, adding an important dimension to the dynamics and stability of group social structure.

Ectoparasites other than ticks can be removed by grooming in primates, and infestation with lice has been looked at recently. Theoretically, social grooming may provide a way that lice are transmitted between partners, increasing infestation, or grooming may remove lice, reducing the infestation. Investigators studying louse infestation and sociality in female Japanese macaques found that females interacting the most with grooming partners had fewer lice than those interacting with fewer partners [26]. Presumably, the effectiveness of grooming in containing the louse load was greater than the transmission effect in increasing louse load.

The grooming picture for primates gets a bit more complicated with a recent study on intestinal parasites in vervet monkeys in South Africa, where investigators found a correlation between group size and degree of hookworm infection [27]. The investigators suggest that the vervet monkeys have some faecal contamination on the fur and skin from contact with faecal-contaminated soil, and a groomer would pick up infective larvae while grooming a partner and ingest the larvae while in the process of grooming.

There are a couple of non-grooming approaches to the control of ticks that are relevant to the discussion on tick control in ungulates. One is a unique adaptation of impala for removing feeding ticks that make it to the ears that are not even accessible to a grooming partner. Impala will quite noticeably accommodate the foraging of oxpeckers on their heads, around their ears, and even hold quite still while oxpeckers forage inside their ears [28]. Ungulates that engage in seasonal migration to areas of high elevation, where the risk of tick exposure is less, appear to groom less frequently than their non-migrating counterparts. In red deer, those that migrate the greatest distance to tick-bare habitats have the lowest tick load [29].

## 4. Avoidance and removal strategies for fleas

Turn next to the household pet that is regularly seen grooming its pelage—the domestic cat. Grooming in the cat is arguably a guide to what one would expect in a wild small cat species. Oral grooming typically occurs in bouts directed in a rostral–caudal direction, and appears to be centrally programmed, as is oral grooming in antelope [30]. The main ectoparasite that affects cats is fleas. Unlike ticks, which move slowly across the body, fleas jump around and are hard to remove. Cats have a tongue with cornified papillae. The papillae serve the cat well in the care of maintaining the fine hair coat, but also in helping to catch fleas.

In an experiment in a flea-ridden environment with over 30 cats, Elizabethan collars were placed on nine cats to prevent oral grooming and flea counts three weeks later were compared with flea counts on nine control cats wearing no collars. The collared cats had double the number of fleas on the control cats [31].

Dusky-footed wood rats make nests in above-ground clumps of branches, usually in a grove of bay trees where they are safe from predators. However, the nest is also an ideal habitat for fleas, where they can feed on the wood rat, hop off and lay eggs, which develop into larvae that then easily re-infect the mammalian host. Wood rats have the unique behaviour of placing fresh bay leaves in the nest area. Investigators wondered if the aromatic bay leaves may be providing a way to avoid what could be an overwhelming flea infestation. This avoidance hypothesis was tested in *in vitro* studies to get precise results. One *in vitro* experiment with adult fleas showed that a measured amount of bay leaf extract was as effective as the traditional *N,N*-diethyl *m*-toluamide (DEET) in repelling fleas [2]. In another *in vitro* experiment with flea larvae, torn bay leaves killed 75% of the larvae, compared with almost none by control plants [32]. This nest fumigation study in wood rats is a mammalian counterpoint to a similar avian phenomenon in starlings, where plants are woven into the nests when young starlings are present. The plants inhibited growth of nest-borne bacteria and also retarded the hatching of louse eggs [33,34].

## 5. Avoidance and removal strategies for intestinal parasites

Very important for mammals living in nature is control of intestinal parasites. Virtually all wild mammals carry some intestinal parasites and they generally manage a modest intestinal parasite load with no evident effects. Parasitic round-worms (helminths) expel ova in faeces, which within a few days (depending upon moisture) hatch into mobile larvae that shortly become infective to new hosts.

For herbivorous ungulates, intestinal parasites are very important because infective larvae are consumed as they crawl up on blades of grass, or other plants, and are eaten by the foraging ungulate. Something has been learned about the immune system and gastrointestinal parasites in domestic ungulates [35,36], and while the immune system is apparently used to a varying degree, there are two behavioural approaches seen in ungulates that play a role in the parasite control. One is to defecate in clumps and the second is to avoid foraging close to faeces, especially when clumped. Several species of farm herbivores have been observed to avoid foraging near conspecific faecal droppings [36]. However, the faeces provide a rich source of nutrients for the forage and one can see in a pasture gazed upon by the ungulates faecal areas surrounded by tall grass that in turn is surrounded by closely cropped grass from the animals foraging near, but not too near, the faeces.

A study involving three wild antelope species (dik-dik, Grants gazelle and impala) in Kenya, serves to illustrate the potential value of defecating in restricted areas or middens and avoiding foraging near the middens. The study confirmed that counts of parasite larvae were greater in the vicinity of the middens than in areas with no faecal droppings or just one clump of faeces. In dik-diks, where foraging behaviour was observed, the animals selectively avoided areas near concentrations of faeces [37].

The generality of defecating in certain areas and avoiding the clumps of faeces in foraging is illustrated in a very different environment by reindeer which use dunging areas and avoid these areas in foraging [38]. For both the antelope and reindeer studied, this strategy of avoiding intestinal parasite reinfection apparently works very well during the dry season but, as shown in both studies, in wet or rainy seasons, when the larvae survive longer, the spread of parasite larvae can be much increased beyond the faecal clumps and the selective foraging is of limited or no value in parasite control. It is important to point out that dunging areas or middens also serve as territorial markers; if the anti-parasite role is lessened at times, the territorial marking role remains.

A question that arises in investigations of ungulate faecal avoidance in grazing is the degree to which the animals might sense a level of intestinal parasite infestation in guidance foraging behaviour. A detailed study on cattle given experimental doses of an intestinal parasite where the animals with the heaviest parasite load were the most cautious in foraging away from faeces supports this perspective [36]. The cattle with the weakest immunological response to the parasites also were those most cautious by foraging away from faeces.

However, in a wild ungulate, the Alpine ibex, that also consistently avoids grazing near faeces [39], the investigator found no relationship in the avoidance behaviour and the animal's level of gastrointestinal parasite infection, indicating that these animals did not seem to sense the level of their parasite infection. The jury would appear to still be out with regard to the relationship between the immunological system, the intestinal parasite load and faecal avoidance in grazing.

The faecal avoidance behaviour is considered a balance between meeting nutritional demands and the degree of risk of acquiring parasites from nearby faeces. The foraging behaviour of the bushbuck in Uganda appeared, initially, to not reflect this balance because bushbucks forage right over faecal-contaminated areas. Upon close examination, it was noted that they do not forage close to the ground level, where parasite larvae would be found, but rather on the stems of grass and other plants at a level above that where the parasite larvae could be picked up [40].

As a final example of herbivores avoiding faecal-borne parasites, a study in Australia on the common eastern grey kangaroo revealed that these animals carry an intestinal parasite load, but manage to keep the load at a tolerable level by avoiding faecal-contaminated forage. While the animals preferred the taller forage with a higher nutrient level, they would not trade a higher parasite risk if the preferred forage was in a contaminated area [41].

The avoidance of faeces and faecal-borne intestinal parasites extends to wild carnivores which also carry an intestinal parasite load. The species-specific roundworms re-infect hosts through larvae that crawl out of the faeces where they can attach to the hair coat of hosts and are groomed off and consumed. Surveys of scats of wolves and wild felids reveal that virtually all carnivores have intestinal parasites [42–46]. One way of reducing the risk of parasite infestations for denning species is the den sanitation strategy of defecating away from the den and rest areas, in some instances in a particular area, making it easy for the group mates to avoid conspecific faeces.

Of course, newborn canids and felids have almost no mobility and cannot eliminate away from the den. They would be at risk of making a faecal mess in the den and re-infesting themselves with intestinal parasites, were it not for their mother's vigilance in quickly consuming their faecal deposits [47]. This maternal coprophagy does not expose the mother to intestinal parasite infection because the parasite ova in the faeces must hatch into larvae before they are infective. An interesting variant of the den sanitation behaviour, which reflects the maternal vigilance in consuming newborn pup faeces, has recently been studied in dogs, where some dogs readily consume faeces of other adult dogs (or their own) that are deposited in their rest areas. This behaviour is overwhelmingly directed at fresh faeces [48]. It was hypothesized that in the ancestral wolf, where faeces might be deposited in the den rest area by an injured or sick wolf, the consumption of recently deposited faeces in the rest area is an adaptive way of keeping the den area free of faecal-borne intestinal parasites because the parasite ova in the fresh faeces would not be infective and the infective larvae would not hatch for a few days.

One might think that species that are primarily arboreal, such as monkeys, would not face the challenges of avoiding faeces because they generally defecate in trees as they move around. However, if the faeces drop on areas to be foraged upon or handled, there is a risk of parasite reinfection. Addressing this risk are observations on the red howling monkey, where it was revealed that they selectively defecate in areas free of underlying branches or vegetation. This selective defecation behaviour, of course, decreases the likelihood of contaminating potential food sources, an observation consistent with the low intestinal parasite load of the monkeys [49].

Mandrills live in dense equatorial rainforests and face intense pressures from intestinal parasites. This is a species that enjoys a good deal of social grooming which undoubtedly helps control ectoparasites as well as having a social function. But mandrills avoid grooming others if they are infected with one of the intestinal protozoan parasites that could be picked up in grooming. When the infected mandrills at the study site were treated for the parasites, they were then groomed more often. Apparently, the parasites change the body odour which group mates sense [50].

## 6. Behavioural defences against pathogens

In contrast to parasites, of which animals living in nature are expected to harbour a load without apparent impact, with pathogenic bacteria or viruses a small dose can lead to a major illness, and accordingly behavioural defences differ from those used with parasites. The immune system is particularly relevant, so the steady state is an immune system activated by gradual exposure to common pathogens in the environment. The pathogens are typically species-specific and animals pick up pathogens in a variety of ways, most notably by being exposed to conspecifics that are sick with a bacterium or virus. For social animals growing up within a group, such as wolves and lions, rejection of non-group conspecifics is an adaptive behaviour from the standpoint of avoidance of pathogenic infections from strangers [1,2].

## 7. Medicine cabinet in the mouth

Aside from exposure to pathogens causing systemic illness from strangers, the environment can harbour opportunistic pathogens that hang around and infect vulnerable animals. For carnivores a main way of dealing with the threat of infections that come about from invasion by surface pathogens is licking behaviour and the protective substances in saliva—‘the medicine cabinet in the mouth’. The range of bactericidal substances in saliva, as studied in dogs and humans, include lysozyme, lactoferrin, leucocytes, lactoperoxidase and immunoglobins [51,52]. For dealing with wounds, one obvious advantage of licking is the washing away of dirt and environmental contaminants. With an open wound, saliva is an all-purpose medicinal ointment. *In vitro* experiments found saliva of dogs to be bactericidal to the common wound contaminants, *Escherichia coli* and *Streptococcus canis* [53]. Saliva also has epidermal growth factors that aid in wound healing [54,55].

The first job for canid and felid mothers that have just given birth is to expose the mammary area and nipples to the newborn so that they can begin nursing and get their first meal of antibody-rich colostrum. However, the lactating mother has, most likely, been exposing her nipples to environmental bacteria from the den floor and adjacent rest areas. The newborn is particularly vulnerable to environmental bacteria as intestinal epithelium is permeable to bacteria for the first 48–72 h [56]. The evolved protective measure of the mothers is to generously lick the nipples starting about a week before the newborn arrives and start to suckle, applying the bactericidal salivary wash which is protective against the common disease-causing pathogens.

Another application for antibacterial saliva in carnivores, rodents and other animals that can easily lick their penises is the apparent prevention of sexually transmissible diseases. Post-copulatory penis licking in rats is so consistent it almost looks compulsory [57]. Two pathogens that have been implicated in rodent genital infections (when they do occur) are *Mycoplasma pulmonis* and *Pasteurella pneumotropica*. *In vitro* experiments reveal rat saliva is effective in killing both of these pathogens [58]. The antibacterial saliva protects males from contracting a venereal disease that may have been harboured by a female they just mated, but also reduces the risk of passing the pathogens on to females they might subsequently mate.

## 8. The cannibalism taboo

Finding adequate food, when relying on regularly capturing prey, is a challenge for a carnivore, whether social or not. Animals may be attracted to scavenge upon dead animals that they could run across. Generally, the fresher the dead carcass, the more appealing and valuable it would be as a food source. Even if the dead animal had died of a pathogenic disease, the animal feeding upon it is not likely to contract the disease—assuming the dead animal is of a difference species—because, in most instances, pathogens are species-specific.

But what about a carnivore running across a freshly dead conspecific? This would appear to be an ideal food source because the animal would be consuming a meal exactly matching the nutritional resources of their own body. But the dead conspecific may have died from an infectious disease that the animal consuming it could contract. The protective behaviour addressing this pathogen risk is referred to as the cannibalism taboo. Carnivores will virtually never feed on the carcass of a recently dead conspecific [59]. But what about a carcass that has decomposed so much it is not recognizable as a conspecific? Using the rat as a model, experiments found that allowing the carcass to decompose, making it unrecognizable to other rats, led to rats eating the carcass [60]. Once the carcass decomposes, saprophytic bacteria take over the carcass and pathogenic bacteria no longer survive. Hence the cannibalism taboo works very well as a pathogen avoidance strategy.

## 9. Pharmacy in the woods: use of medicinal herbs for parasites and pathogens

Herbal medicine, as practised in traditional human cultures, and long before modern medicines existed, has been extensively studied. Several, or even most, modern medicines have an origin in ancient herbal remedies that, at least sometimes, removed parasites or pathogens and avoided major infections. The plant-derived medicinal substances, referred to as secondary plant compounds, are effective in protecting the plant from foraging insects, parasites and pathogens. It may be simply a coincidence that they have the medicinal effects for animals [61].

The most convincing example of the use of herbal medicine for pathogens in mammals was an observation of a sick chimpanzee that was seen extracting, and chewing upon, the bitter pith of a *Vernonia amygdalina* plant, which is known to have antimicrobial effects [62]. Follow-up observations suggested that the chimpanzee quickly recovered from the illness. No specifics were given with regard to the pathogen. A recent study on woolly spider monkeys (*Brachyteles arachnoides*) revealed that the diet of this primate includes plant species with demonstrated medicinal properties and that these were used in a medicinal way by humans in areas surrounding the park [63].

Information on the specific constituents of plant parts that convey the therapeutic effects has come from work on animal models in the laboratory. Various medicinal herbs have been shown to have one or more of the following properties: anti-inflammatory, antimicrobial, immunomodulatory, analgesic [61]. The anti-inflammatory property is the most common, and it seems logical that in nature reducing inflammation will allow the animal to more easily move about, obtain food and water and/or care for young. The antimicrobial constituent would, of course, be adaptive in reducing or eliminating infections. Such antimicrobial constituents are broad spectrum and would be valuable in several types of infections, albeit not as specific as modern antibiotics. The immunomodulatory constituents appear to enhance the immune system and aid in helping the immune system rid the body of pathogens. Analgesic constituents may help in easing the pain associated with an infection.

The constituents mentioned are rarely found alone. Usually, an anti-inflammatory constituent would be found along with an antimicrobial or immunomodulatory constituent. Thus, the medicinal plant part is broad spectrum. The secondary plant compounds with medicinal effects usually have a bitter or astringent taste, which apparently protects the plant from being grazed upon by herbivores.

For primates, including humans, the bitter plant part will not be sought out as food. But it is hypothesized that when ill, there is a lowering of the threshold for bitter, and even an evolved predisposition to seek out a bitter-tasting plant part that has some probability of being effective for the animal's illness [61]. The therapeutic herb sought out by ill chimpanzees, *V. amygdalina*, is bitter tasting, indicating that the sick individual was experiencing illness and possibly sought out the ‘bitter pill’.

A different angle of animal herbal medicine is the use of the ‘pharmacy in the woods' for anthelminthics to expel intestinal parasites from the intestinal tract. Studies on chimpanzees [64] as well as bonobos [65] reported the frequent swallowing of whole leaves of local plants (e.g. *Manniophyton fulvum*) with the effect of purging of intestinal parasites. Wild chimpanzees eat leaves from a variety of plants that pass through the intestinal tract whole. In some instances, the plant material increases intestinal motility that, which then purges the intestinal tract of nematodes. Sometimes in scats the leaves are even seen wrapped around worms that have been expelled. There can be seasonal differences in the risk of nematode infections in chimpanzees, and when one particular nematode increased in the study site in Tanzania, the swallowing of whole leaves increased, indicating that the animal sensed an increased parasite burden [66].

**Figure 4.**
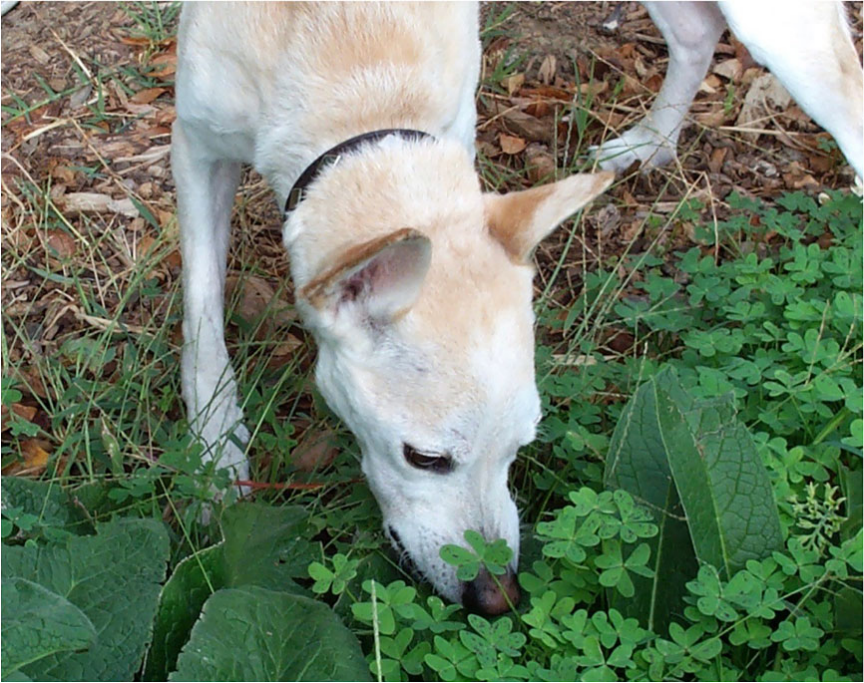
Plant-eating by a dog. Dogs typically show no signs of illness and do not vomit after plant-eating [72]. (From [2], copyright B.L.H.). (Online version in colour.)

The most common use of plants as protection from intestinal parasites is seen in canids and felids (figure 4) commonly ingest non-nutritional plants, especially grass (figure 4). Plant-eating is seen by multitudes of dog and cat owners as a reflection of the behaviour inherited from their wild ancestors. Evidence that regular consumption of non-digestible plant material occurs in wild canids and felids is that grass and leaves have been found in a range of 2–74% of scats and stomach content samples of wolves and cougars [67–70]. In his field studies, the noted wolf biologist, Murie, described seeing leaves of grass wrapped around expelled intestinal worms in wolf scats [71], suggesting that plants purged or expelled intestinal worms (figure 4).

Just as humans cannot feel worms in their intestines, dogs and cats (and their wild ancestors and relatives) presumably cannot feel, or otherwise know, whether or not they are infected with worms, aside from a vague stuffed feeling. The evolution of regular plant-eating by canids and felids is arguably an adaptive ongoing strategy for maintaining the intestinal parasite infection at a low to moderate level.

In veterinary clinical practice, the traditional explanation for plant-eating in dogs and cats is that there is a dietary deficiency or that plant-eating is a way of inducing vomiting. In two broad-ranging Web-based surveys of thousands of dog and cat owners, it was found that the great majority of dogs and cats appeared normal before and after eating plants and did not vomit [72,73] (figure 4). An important finding was that animals under 1 year of age ate plants much more frequently than older ones, the explanation being that the young are more vulnerable to the cost of intestinal parasites, and hence have an evolved tendency to eat plants more frequently.

The use of medicinal compounds in plants can also extend to ungulates as they graze on plants. Observations have been made on domestic ungulates that were reported to self-medicate on plants in association with an apparent ailment. One report interviewed pastoralists in Uganda about consumption of known medicinal plants, using the specific criteria for self-medication: (i) the animal had an obvious ailment; (ii) the animal was consuming a plant or plant part that it rarely did when healthy; (iii) there was subsequent improvement in the animal's signs related to the ailment and (iv) the animal no longer continued the self-medication [74]. This study reported 87 instances involving 50 self-medicating behaviours in various ungulates meeting these criteria. Another study found that goats infected with 10 000 larvae of mixed gastrointestinal nematodes increased their preference for an anti-parasitic shrub [75].

A review of several ruminant self-medication studies raises several relevant questions. How do they experience malaise during a parasitic infection? How do they experience relief after consuming an anti-parasitic plant? How do they identify medicinally active plants? When they get sick do they preferentially forage on anything that tastes or smells unpleasant, such having as a bitter taste? Do they learn from other animals? Do they learn by trial and error? Does the unpleasant taste of medicinal foliage become more acceptable to a sick animal [76]?

## 10. Conclusion

In surveying the broad picture of how animals stay healthy and thrive in nature several points come to mind. Animals are generally not free of parasites either on the body surface or in the intestinal tract, but the parasite load is manageable by the strategies discussed. The avoidance behaviours are specific to the most important threats of the specific animal species. The strategies for controlling both parasites and pathogens in mammals living in nature include: avoiding external parasites, such as flies by fly switching; removing ticks, fleas and lice by grooming; using nest fumigation to repel and kill fleas; defecating in certain areas, and avoiding faeces in foraging; practising den sanitation to avoid intestinal parasites; showing the cannibalism taboo to avoid systemic infections from dead conspecifics; using the medicine cabinet in the mouth to remove surface pathogens; and using the pharmacy in the woods to get plant parts with medicinal properties, including natural worm-purging anthelminthics. Although mammals are emphasized in this paper, behavioural strategies have been identified in other vertebrate and invertebrate species that illustrate the variety of strategies used by animals living in nature to control the risk of infection from parasites and pathogens. Given the focus in modern society on human and veterinary medicine, and the never-ending search for ways to control pathogens and parasites that have rapidly evolved resistance to our defensive antibiotics and other treatments, we might profit from learning more about how animals do so well in nature without access to modern medicine, and stay healthy and thrive.

## Data accessibility

This article has no additional data.
